# Violacein: Properties and Production of a Versatile Bacterial Pigment

**DOI:** 10.1155/2015/465056

**Published:** 2015-08-03

**Authors:** Seong Yeol Choi, Kyoung-hye Yoon, Jin Il Lee, Robert J. Mitchell

**Affiliations:** ^1^School of Life Sciences, Ulsan National Institute of Science and Technology, 50 UNIST-gil, Eonyang-eup, Ulsan 689-798, Republic of Korea; ^2^Division of Biological Science and Technology, College of Science and Technology, Yonsei University, 1 Yonseidae-gil, Wonju, Gangwon-do 220-710, Republic of Korea

## Abstract

Violacein-producing bacteria, with their striking purple hues, have undoubtedly piqued the curiosity of scientists since their first discovery. The bisindole violacein is formed by the condensation of two tryptophan molecules through the action of five proteins. The genes required for its production, *vioABCDE*, and the regulatory mechanisms employed have been studied within a small number of violacein-producing strains. As a compound, violacein is known to have diverse biological activities, including being an anticancer agent and being an antibiotic against *Staphylococcus aureus* and other Gram-positive pathogens. Identifying the biological roles of this pigmented molecule is of particular interest, and understanding violacein's function and mechanism of action has relevance to those unmasking any of its commercial or therapeutic benefits. Unfortunately, the production of violacein and its related derivatives is not easy and so various groups are also seeking to improve the fermentative yields of violacein through genetic engineering and synthetic biology. This review discusses the recent trends in the research and production of violacein by both natural and genetically modified bacterial strains.

## 1. Natural Violacein-Producing Strains and Their Locales Are Quite Diverse

**Figure 1:**
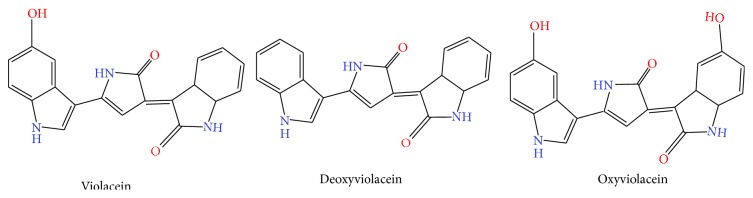
Structures of violacein, deoxyviolacein, and oxyviolacein showing either the presence or the lack of the hydroxyl groups. The structures were prepared using ChemDoodle 2D sketcher (http://web.chemdoodle.com/demos/sketcher).

As a bisindole, violacein (Figure 1) is produced by diverse genera of bacterial strains, including* Collimonas* [1],* Duganella* [2],* Janthinobacterium* [3–5],* Microbulbifer* sp. [6], and* Pseudoalteromonas* [7–9]. These violacein producers are varied phylogenetically and so are the locales from which they have been isolated. As shown in Table 1, these include quite a selection of environs as these bacteria have been found associated with the surfaces of sea sponges [7] and the rhizosphere of olive groves [10] and even within glaciers [4, 11, 12]. Perhaps the best known genus, however, is* Chromobacterium* [13, 14], which includes the strain* C. violaceum* [15].

## 2. Violacein as an Indicator of Quorum Sensing

In most of violacein-producing bacterial strains isolated from nature, this bisindole is a secondary metabolite that is associated with biofilm production [5]. Moreover, its production within* C. violaceum* and other strains is regulated by quorum sensing mechanisms [16]. Because it is easy to visualize, violacein production by* C. violaceum* has become a useful indicator of quorum sensing molecules and their inhibitors [17–20].

Secondary metabolites often serve functions other than the bacteria's immediate needs in growth and propagation. Many of these molecules are biologically active, and some have toxic properties to competing species giving the bacteria a competitive advantage. Because of these activities, many secondary metabolites have been found to have pharmacological properties and, thus, are of interest for clinical use. In the first half of this review, therefore, we will review some of the biological functions, clinical significance, and mechanisms of action of violacein.

## 3. Violacein's Function in Nature-Predator Defense?

As noted above, violacein-producing bacteria encompass various genera and are found in all types of natural environments, from marine to freshwater and soil environments (Table 1). For this reason, it is rather difficult to pinpoint violacein's main biological or ecological role. One possible commonality is that violacein producers are generally sessile bacteria which makes them more vulnerable to predation [6]. This leads to the idea that violacein serves as some sort of defense mechanism for its producing strain or that it provides these sessile bacteria some competitive advantage.

Consistent with this, violacein was shown to have antibacterial properties particularly towards Gram-positive bacterial strains [36, 37]. One strain in particular that has received most of the recent attention is* Staphylococcus aureus*, in part due to its status as a multidrug resistant pathogen [37–40]. Two studies showed that crude violacein was capable of inhibiting* S. aureus* growth at concentrations between 5.7 and 15 mg/L, or approximately 17 to 43 *μ*mol/L [38, 40].

Violacein's biological activity is not limited to prokaryotes, however, as it is also known to have negative effects on bacteriovorus protozoans and metazoans (Table 2). Matz et al. tested purified violacein and violacein-producing biofilms with various protozoan species that include flagellates, ciliates, and amoeba [6]. They showed that, when exposed to violacein, the bacteriovorus amoeba* Acanthamoeba castellanii* displays decreased feeding accompanied by morphological changes, such as cell rounding. Further observations, such as increases in caspase-3-like activity and TUNEL assay studies, suggest that these predacious microbes are dying via an apoptotic-like cell death [41].

Like many protists, the soil nematode* C. elegans* is a bacteriovorus predator that ingests a wide variety of microorganisms [42]. Although* C. elegans* is best known as a premier genetic model organism that has propelled such important discoveries as RNAi, microRNAs, and programmed cell death [43–45], it is also an excellent tool to understand violacein's biological activity on bacterial predators. For example,* C. elegans* fed* E. coli* as a prey strain grows normally and healthily in the laboratory. However, two strains of bacteria that produce violacein,* Janthinobacterium* sp. HH01 and* C. violaceum,* were shown to be toxic to* C. elegans* [27, 46]. In particular HH01 exposures resulted in developmental problems in juvenile worms, as well as behavioral changes and a rapid mortality in adult worms. Interestingly, an exposure to a mutant HH01 strain that lacks violacein and tryptophan production did not induce severe toxic effects, indicating that the responses originated with the bisindole. As further confirmation of this,* E. coli* carrying HH01 vioA-E genes that produce violacein resulted in accelerated death, albeit at a slower rate than in HH01. These experiments affirm that violacein is likely toxic to the bacterial predator* C. elegans*.

In addition to its apparent roles in protecting the bacterium from predation, violacein seems to have other ecological functions that may provide advantages for the bacteria that produce it. For instance, violacein-producing* Janthinobacterium* forms biofilms and resides on the skins of frogs and salamanders [24, 47]. In return, the violacein produced provides antifungal protection for its amphibian hosts, increasing their survival.

## 4. Potential Clinical Uses of Violacein

These functional studies portray the secondary metabolite violacein as a toxic sentinel guarding against diverse potential bacterial predators and other competitors. However, such a broad cellular toxicity may also prove to be useful as a therapeutic against various pathogenic and endogenous cellular insults. Consistent with this, many pharmacological properties have been attributed to violacein.

Violacein has strong antibacterial effects making it a promising candidate as an antibiotic. Moreover, when administered in combination with other antibiotics, the impact is more effective in fighting bacteria than the use of antibiotics alone [48]. This is of particular interest in light of recent antibiotic-resistant strains of pathogenic bacteria, such as MRSA. Also, the antiprotozoan properties of violacein could be exploited to treat diseases in humans, such as in malaria and leishmaniasis [25, 26].

The most studied clinical use of violacein, however, is it being a potential cancer therapeutic. Violacein has been tested against various cancer cell lines (Table 3), where it has shown cytotoxicity at IC50 values that mostly range in the submicromolar concentrations. The effects of violacein were also shown to be specific for the cancer cell line tested as two colorectal cancer cell lines, Caco-2 and HT-29, were differentially susceptible to violacein [31]. Since violacein is cytotoxic towards noncancer cells as well [28, 34], the critical factor for its clinical use against cancer is that it is more toxic to cancer cells than to normal cells. One study demonstrated that violacein induced apoptosis in HL60 cells (IC50 = 700 nM), a cancer cell line used as a model to study myeloid leukemia. However, normal lymphocytes were unaffected at the concentrations tested, further asserting violacein's use as a putative cancer therapeutic [34].

Violacein clearly has toxic effects on cultured cancer cells, that is, within* in vitro* tests. Its ability to attenuate cancer growth* in vivo* was also tested recently in the Ehrlich ascites tumor (EAT) mouse model [35]. Daily intraperitoneal injections of 0.1 *μ*g/kg violacein significantly increased the survival rate of the mice, while no adverse effects were observed in mice receiving higher doses of up to 1 mg/kg.

## 5. Biological Mechanism of Violacein's Effects

Even though violacein is a promising agent as an antibiotic and a treatment for cancer, the biological mechanisms behind these actions remain elusive. Before we can stamp violacein as a natural therapeutic for numerous kinds of diseases, it is imperative to understand its mechanisms of action at the cellular and molecular levels.

Several groups have begun this and many of the studies performed in cancer cell lines report increased activity in indicators of apoptosis-related markers, such as increases in the reactive oxygen species (ROS) and the activation of caspases [31, 32, 34, 35]. However not all cancer cell lines respond to violacein and the reason for the selectivity is not well understood. Moreover, among the leukemia cell lines tested in the literature, violacein showed selective cytotoxicity against HL60 and TF1 (Table 3), but the pathways that lead to cell death were very different in the two cells. In HL60 cells, an exposure to violacein led to phosphorylation of p38 MAP kinase, upregulation of the NF*κ*B pathway, and activation of caspases [34]. It was also found that TGF*α* receptor activation was required for these downstream effects. TF1 cells, on the other hand, did not seem to follow the canonical apoptotic pathway as treatment with inhibitors of proapoptotic caspases did not prevent cell death [33].

Conclusions on violacein's mechanism of action are clearly scant at this point. However, the fact that violacein has cytotoxic effects on such a wide variety of organisms and cells hints at a common target or pathway. Studying the effect of these bisindoles at the genetic level on model eukaryotic organisms, such as* C. elegans*, will help in elucidating its mechanism of action and enrich our knowledge of violacein as a clinical therapeutic. Owing to its versatile activity against many human ailments and infectious agents, however, it is not surprising that this bisindole has garnered more attention recently from the scientific community. One factor that may contribute to reducing violacein's application, though, is its relatively low level production by natural strains. Consequently, the latter half of this review will be primarily given to the discussion of current research into the production and purification of violacein and its related derivatives.

## 6. Production of Violacein by Natural Host Strains

Since violacein is produced naturally by various bacterial species, the use of these strains for its production seemed like a clear choice. However, many factors were found to influence the yields, including the agitation and aeration [7], the inoculum size [2, 10], and the nutrients available [2, 10, 49, 50]. It should be noted that the violacein concentrations reported within many of the articles are based upon the extinction coefficient as determined by the authors using spectrophotometric analyses, with extinction coefficient values ranging between 10.955 and 74.3 L/(g-cm) in the literature [2, 49, 51, 52]. A recent article by Rodrigues et al. highlighted the discrepancy caused by spectrophotometric-based determinations of violacein and deoxyviolacein concentrations and stated that this could result in violacein concentrations that are inflated by as much as 680% [51]. To address this in their study, therefore, Rettori and Durán (1998) relied on HPLC measurements, a technique which was proposed in an earlier study to be used in parallel with NMR, UV-Vis spectroscopy, and mass spectroscopy when characterizing violacein and its production by bacterial strains [52]. Consequently, to avoid any potential confusion to the readers, this report will provide the violacein and deoxyviolacein concentrations reported and state whether they were determined via HPLC or with an extinction coefficient.

One group applied response surface methodologies (RSM) to identify the best conditions to produce violacein with* C. violaceum* [49]. They initially analyzed 16 variables but eventually limited them to three—glucose, tryptone, and yeast extract. While the latter two improved both the cell mass and violacein yields as they were increased, glucose was found to be negatively correlated with the violacein yields, and limiting its addition to the culture was advantageous. Using this technique, they were able to increase the dry cell weight (DCW) from 7.5 to 21 g/L and the violacein yields from 170 to 430 mg/L with an extinction coefficient of 56.01 L/(g-cm). Although there was a significant improvement in the volumetric productivity of violacein, defined as the mg of product per liter of culture, it should be noted that this does not represent a greater specific productivity of this bisindole, that is, mg product per gram of cells, as the cell mass increased by 2.8-fold while the violacein concentration increased by only 2.5-fold. Yet, similar protocols could potentially be employed with other strains to increase the cell density and, thus, raise the volumetric productivity.

A more recent study using RSM with another violacein-producing strain,* Pseudoduganella* sp. B2, previously* Duganella* sp. B2, found similar results as the concentration of beef extract used was a major impetus for violacein production [2]. They also identified the culture pH and the concentrations of tryptophan and potassium nitrate as major players influencing the final violacein yields. The importance of tryptophan is not surprising as this is the precursor for violacein, while the impact of nitrate was thought to be related to nitrogen source availability for the growing bacterial cultures. Under the optimal conditions found, the authors claimed* Duganella* sp. B2 was capable of producing 1.6 g/L crude violacein, typically referring to the naturally produced mixture of violacein and deoxyviolacein. This value is under scrutiny, however, as the extinction coefficient used (10.955 L/(g-cm)) is the lowest reported in the literature [2, 49, 51, 52]. 

## 7. Production of Violacein within* E. coli* and Other Heterologous Hosts

Since the genes required for the production of violacein are known to exist within a single operon, that is,* vioABCDE* [53], many groups have sought to clone and express these within other bacterial hosts, including* E. coli* [8, 51, 53]. The Pemberton group has focused on plasmid stability, an issue when trying to generate bacterial products in long term and in nonnatural hosts. They found, for instance, when the violacein gene cluster was cloned into pHC79, a cosmid vector, that it was unstable and was lost in as much as 60% of the bacterial population when grown for 15 generations in the absence of antibiotic pressure [54]. They claimed that the same was true when they expressed the* vioABCDE* operon in a pUC18 vector but were able to generate a stable construct using a broad host range IncP plasmid [54, 55]. This plasmid, pPSX-Vio^+^, was stable without antibiotics for more than 100 generations [54], making it a potentially useful tool for the production of violacein.

In a subsequent study, they found that the amount of violacein produced by* E. coli* was dependent upon the host, with* E. coli* strain JM109 producing 3.9-fold more violacein than* E. coli* strain DH5*α* when harboring the same plasmid [55]. The production of the alpha amylase protein, AmyA, from* Streptomyces lividans* was likewise found to be better in JM109 than DH5*α*, demonstrating that this phenotype was characteristic of JM109 and not due to the violacein genes. The authors attributed better results with JM109 to the genetic differences between the host strains but did not delve deeper into the mechanisms underlying this phenotype. However, they did identify a mutation within the plasmid which led to a further enhancement in the violacein yields [55]. This mutation, which the authors designated as* opv*-1 (overproduction of violacein), results from the deletion of a single nucleotide within the region upstream of the* vioA* gene, leading to a 4.2- and 2.9-fold enhanced violacein yield from* E. coli* strains DH5*α* and JM109, respectively.

A more recent study sought to engineer* E. coli and* its metabolic pathways to improve the violacein yields [51]. For this, the authors overexpressed the genes related to tryptophan production and knocked out several genes and pathways which would detract the carbon flux away from this amino acid. The engineered* E. coli* strain, TRP11, produces about 20 *μ*mol tryptophan per gram DCW (gDCW). By comparison, the control wild-type strain only produced about 0.3 *μ*mol tryptophan/gDCW, representing an increase in the tryptophan concentration of more the 60-fold. They next introduced the* vioD* gene into the chromosome of this strain and a plasmid expressing the* vioABCE* genes. Performing fed-batch fermentations over a 12-day period with this strain, which they designated as Vio-4, they were able to generate 710 mg/L violacein at more than 99% purity, demonstrating that* E. coli* can be used to produce high level concentrations of this bisindole specifically.* E. coli*, however, has not been the only strain to have been used as a host to produce the bisindole violacein. Both* Citrobacter freundii* and* Enterobacter aerogenes* have also been used with positive results [56].

## 8. Deoxyviolacein and Oxyviolacein

Although violacein has received most of the attention, it is not the only compound being produced by the VioA, VioB, VioC, VioD, and VioE proteins within these bacterial hosts. A recent article that describes the various products in detail was recently published and lists over a dozen different compounds that have been produced in tests with various strains, mutants, and cell-free extracts [13]. Perhaps the best known derivative produced by the violacein biosynthetic pathway, however, is deoxyviolacein. In* J. lividum*, for example, deoxyviolacein (Figure 1) is also being produced, albeit at a lower level than violacein [57]. Similar results were also seen with* Duganella* sp. B2 where the production of deoxyviolacein was lower than that of violacein, as measured by HPLC [58]. The percent deoxyviolacein within the crude violacein extracts obtained from the natural bacterial hosts is typically around 10~20%, with the vast majority being violacein. This is also true for commercially available violacein from Sigma-Aldrich, which is prepared using* J. lividum* and certified as at least 85% violacein based upon HPLC analysis.

Deoxyviolacein, which lacks a hydroxyl group (Figure 1), can be produced by omitting the VioD protein. Using a recombinant* Citrobacter freundii* carrying a plasmid with a knock-out in the* vioD* gene, a recent study by the Xing group reported on the high level production and characterization of deoxyviolacein [58], with an emphasis given to the differences between violacein and deoxyviolacein. Their study showed, for example, that the photostability of deoxyviolacein was slightly better than violacein in tests with either natural or UV light. Moreover, in 24-hour toxicity tests with HepG2 cell lines, violacein and deoxyviolacein were both found to be toxic. However, the impact of violacein was dose-dependent as greater additions of this bisindole led to greater concomitant losses in the HepG2 viability. In contrast, the toxicity of deoxyviolacein was not dose-dependent but led to fairly consistent and stable losses in the viability over a range of concentrations (0.1–10 *μ*M). The difference between these two bisindoles was even more pronounced when the viability was determined after 48 hours.

Several groups have also shown that the proteins responsible for the biosynthesis of violacein are not strict for their typical substrate, tryptophan, as 5-hydroxytryptophan can also be used to produce another derivative, oxyviolacein (Figure 1) [59, 60]. In contrast to deoxyviolacein which lacks a hydroxyl group when compared with violacein, oxyviolacein boasts an additional hydroxyl group. Likewise, as the loss of the hydroxyl group in deoxyviolacein reduced its efficacy against* S. aureus*, the presence of this extra hydroxyl group within oxyviolacein was found to increase the potency against these human pathogens [61].

## 9. Conclusions

This review presents current research trends regarding the study and production of the bacterially produced bisindole violacein and several derivatives. As a secondary metabolite, violacein has been found to possess a wide variety of biological activities, including anticancerous properties. These characteristics have led to renewed interest in this compound and its production by both wild-type and recombinant bacterial strains. As presented in this report, the production and characterization of violacein are not without their own obstacles and struggles, and much work still needs to be done. This is particularly true regarding the mode of activity of violacein which needs to be studied more in depth. Current trends in molecular genetics are aiding in this as researchers are now capable of engineering bacterial host that can overproduce this bisindole within fermentations. As work with this compound and its derivatives progresses, it is anticipated that violacein will become more readily available for the scientific community and clinical studies.

## Figures and Tables

**Table 1:** Some violacein-producing strains and the locales from which they were isolated.

Strain	Locale	Reference
*Chromobacterium violaceum *	River Waste water treatment plant	[14] [21]
*Collimonas* sp.	Arctic coastal waters	[1]
*Duganella* sp.	Agricultural soils (olive)	[10]
*D. violaceinigra *	Forest soils	[22]
*Janthinobacterium lividum *	Glacier	[4]
*J. svalbardensis *	Glacier	[11]
*Pseudoalteromonas* sp.	Deep sea waters (320 m)	[23]
*P. luteoviolacea *	Sea sponge surface	[7]

**Table 2:** Eukaryotic organisms in which violacein was shown to produce negative effects.

Type	Organism	Description	Reference
Fungi	*Batrachochytrium dendrobatidis *	Infects amphibians	[24]

Protozoa	*Rhynchomonas nasuta *	Flagellate	[6]
	*Tetrahymena *sp.	Ciliate	[6]
	*Acanthamoeba castellanii *	Amoeba	[6]
	*Leishmania amazonensis *	Causative agent of leishmaniasis	[25]
	*Plasmodium falciparum *	Causative agent of malaria in humans	[26]
	*Plasmodium chabaudi chabaudi *	Causative agent of malaria in mice	[26]

Nematode	*Caenorhabditis elegans *		[27]

**Table 3:** List of cell lines tested against violacein.

Cell line	Cell type	Organism	Notes	Reference
V79	Fibroblast-like cell line from lung tissue	Chinese Hamster		[28]
FRhK-4	Fetal kidney	Monkey		[29]
Vero	Kidney	Monkey		[29]
MA104	Kidney epithelial cells	Monkey		[29]
Hep2	Hela-derived	Human		[29]
92.1	Uveal melanoma	Human		[30]
OCM-1	Choroidal melanoma	Human		[30]
NCI-H460	Non-small-cell lung cancer	Human		[28]
KM12	Colon cancer	Human		[28]
Caco-2	Heterogeneous epithelial colorectal adenocarcinoma	Human		[31, 32]
HT29	Colorectal adenocarcinoma	Human		[31]
HCT116	Colorectal adenocarcinoma	Human		[32]
SW480	Colorectal adenocarcinoma	Human		[32]
DLD1	Colorectal adenocarcinoma	Human		[32]
TF1	Erythroleukemia	Human		[33]
K562	Lymphoma	Human	N/C^a^	[34]
U937	Chronic myelogenic leukemia	Human	N/C^a^	[34]
HL60	Promyelocytic leukemia	Human		[34]
MOLT-4	Acute lymphoblastic leukemia	Human		[28]
EAT	Ehrlich ascites tumor	Mouse	*In vivo * ^b^	[35]

^a^No cytotoxicity observed.

^b^Both *in  vitro* and *in  vivo *tests were performed.
